# F113. IMPACT OF NEIGHBORHOOD CHARACTERISTICS ON PARANOIA, LONELINESS, AND PERCEIVED REJECTION IN A TRANSDIAGNOSTIC SAMPLE WITH PSYCHOSIS

**DOI:** 10.1093/schbul/sby017.644

**Published:** 2018-04-01

**Authors:** Christina Savage, Cristina Garcia, LeeAnn Shan, Alexandra Andrea, Melanie Bennett, Jack Blanchard

**Affiliations:** 1 University of Maryland; 2 University of Maryland School of Medicine

## Abstract

**Background:**

Paranoia is unsubstantiated thinking that others want to cause harm, and it exists on a spectrum ranging from suspicion to delusions both in the general population and in individuals with psychosis (Freeman et al., 2011; Freeman, 2016). Paranoia is interpersonal in nature, and research has shown that individuals with paranoid delusions use person attributions more than depressed or healthy controls during a task with interpersonal vignettes (Bentall, Kaney, & Dewey, 1991). Therefore, it is important to explore how other interpersonal or social factors may affect paranoia. Neighborhood characteristics, such as reduced social cohesion and crime, perceived rejection, and loneliness have been associated with paranoia (Lamster, Nittel, Rief, Mehl, & Lincoln, 2017; Newbury et al., 2017; Wickham, Taylor, Shevlin, & Bentall, 2014). However, it is still unclear whether these factors affect paranoia independently or have a more additive influence. Some researchers have proposed that perceived rejection and loneliness reduce community engagement and the size of social networks in this population (Cechnicki & Wojciechowska, 2008; Kidd et al., 2016), but others have not supported these findings (Trémeau et al., 2016). The current study will try to clarify the literature by exploring the associations between neighborhood characteristics, social factors (namely, perceived rejection and loneliness), social network, and paranoia.

**Methods:**

The current study will examine how paranoia correlates with neighborhood characteristics, loneliness, perceived rejection, and social network size in a transdiagnostic sample with psychosis. We will utilize the Neighborhood Environment Scale (Mujahid et al., 2007) to assess social cohesion, safety, violence, and activities with neighbors within participants’ residencies. We will use the Paranoid Thought Scales (Green et al., 2008) to assess paranoid ideation and the Adult Social Relationship Scales (Cyranowski et al., 2013) to assess perceived rejection and loneliness over the past month. In addition, we will use the Social Network Index (Cohen et al., 1997) to investigate the correlation between participants’ social network and paranoia, social rejection, and loneliness.

**Results:**

Preliminary results (N = 13) indicate a significant correlation between paranoia and perception of neighborhood social cohesion (r = -0.57, p < 0.05). In addition, loneliness (r = 0.60, p < 0.05) and perceived social rejection (r = 0.52, p < 0.05) were the largest correlates of paranoia. We will conduct formal analyses with a larger N to further explore these and other associations.

**Discussion:**

Discussion will be included in the poster after more data has been collected.

